# Late preterm infants’ growth and body composition after discharge

**DOI:** 10.1186/1824-7288-40-S2-A27

**Published:** 2014-10-09

**Authors:** Paola Roggero, Maria Lorella Giannì, Nadia Liotto, Pasqua Piemontese, Fabio Mosca

**Affiliations:** 1 NICU, Department of Clinical Sciences and Community Health, Fondazione IRCCS Cà Granda Ospedale Maggiore Policlinico, Università degli Studi di Milano, Milano, Italy

## Background

The proportion of late preterm births has markedly increased during the past two decades, accounting for 70% of preterm births [1]. There is evidence that monitoring not only the quantity but also the quality of growth, in terms of body composition changes, may play an important role in gaining further insight into the relationship between birth weight and time in utero on early growth pattern and future health [2]. To our knowledge, data regarding the early dynamic features of growth and body composition changes of late preterm infants are scarce [3-5]. The aim of this study was to compare growth and body composition of late preterm infants to that of very preterm and full term infants.

## Materials and methods

Observational longitudinal study. Forty-nine late preterm infants and 63 adequate for gestational age very preterm infants were included in the study. Forty healthy, full-term, breast-fed infants were enrolled as a reference group. Anthropometric parameters and body composition by an air displacement plethysmography system were assessed at term, at 1 and 3 months of corrected age in all groups.

## Results

Basal characteristics of the study population are shown in table 1.

**Table 1:** Basal characteristics and anthropometric and body composition parameters at term and at 3 months of corrected age of the study population

	Full term infants	Late preterm infants	Very preterm infants
**Gestational age (wks)**	38.8 ± 1.4	35.3 ± 0.75	29.1 ± 2.1
**Birth Weight (g)**	3074 ± 409	2496 ± 330	1202 ± 238
**Birth length (cm)**	49.3 ± 2	44.8 ± 1.7	37.2 ± 3.5
**Birth head circumference (cm)**	34.2 ± 1.17	31.6 ± 1.2	29.07 ± 2.1
**Weight 40 wks (g)**	3074 ± 409	3396*° ± 390	3015 ± 403
**Fat free mass 40 wks (g)**	2794 ± 358	2837° ± 255	2459 ± 320
**Fat mass 40 wks (g)**	280 ± 106	559# ± 196	565 ± 168
**Weight 3 mo (g)**	5978 ± 722	6197° ± 589	5557 ± 669
**Fat free mass 3 mo (g)**	4345 ± 484	4500^ ± 390	4157 ± 461
**Fat mass 3 mo (g)**	1632 ± 355	1672^ ± 348	1405 ± 362

*late preterm vs full term p=0.001

° late preterm vs very preterm p< 0.001

# late preterm vs full term p<0.001

^ late preterm vs very preterm p= 0.005

Late preterm infants showed higher weight at term than full term and very preterm infants (table 1). Length (49.5 vs 47.5 cm; p< 0.0001) and head circumference ( 35.2 vs 34.4 cm; p= 0.004) values were also bigger in late preterm infants at term than in very preterm infants. At 3 months of corrected age no significant difference in anthropometric parameters was found between late preterm and full term infants, whereas weight of late preterm infants was higher than that of very preterm infants.

With regard to body composition, fat mass at term of late preterm infants was similar to that of very preterm but significantly higher than that of full term. Fat free mass at term was not different between late preterm and full term infants while very preterm infants showed the lowest value.

At 3 months of corrected age, late preterm infants reached a similar body composition to full term infants, whereas very preterm infants still had the lowest values of weight, fat free mass and fat mass (table 1).

## Conclusions

The present findings demonstrate that late preterm infants have an altered body composition at term corrected age in terms of high adiposity. Potential metabolic implications of these results need to be investigated.
